# Abstracts from Foreign Journals

**Published:** 1896-02

**Authors:** 


### ABSTRACTS FROM FOREIGN JOURNALS.

Professor Horday on "New Instruments" before the Central Veterinary Medical Society, London, England: "I have two ideas, which I believe to be new, and which I desire to place before you this evening. The first is a speculum, or gag, designed to open the mouth of a dog, pig, or other small animal. The method of application is as follows: Secure the patient firmly by the aid of an assistant or on an operating table, and place the closed gag in the animal's mouth, tightening the straps which pass over the upper and lower jaws. When ready to open the mouth, turn the two screws together equally and steadily, and the jaws will be slowly forced apart. It is made entirely of metal and can be kept clean and aseptic; the metal used, termed 'Dorion,' is beautifully smooth and soft to the touch (thus dispensing with rubber coverings, which soon become injured and dirty), and will not corrode or rust in any way. By means of having the rings to slide along the bar it can be made to fit any size of dog or pig. I have given it a thorough test on all breeds, and have found it of great service when wishing to examine or extract the teeth, to remove bones, needles, or other foreign bodies from the mouth.

"The second idea is that of a thermometer marked with the average temperature of the various domesticated animals. For some long time past I have had an idea that such a design would be of service to veterinary surgeons. Those of us who live in large towns are occasionally called in to see animals, such as sheep, pigs, etc., which come more commonly in the range of agricultural practice. If we used the thermometer as an aid to diagnosis, we might pardonably have forgotten that different animals have different body temperatures. Personally, it was for a long time a source of difficulty to me to recollect the normal figures unless I was constantly taking them.

"For the purpose of obtaining these averages I have been engaged in making observations in different parts of England since March last in order to notice whether extreme of weather, different diets, different districts, confinement or liberty, exercise, excitement, or any other extrinsic cause altered in any way the rectal temperature. The results have been of exceeding interest. I have utilized almost 800 animals and birds, all apparently in good health, rejecting any that showed the slightest sign of disease, either external or internal. In some of them I have been able to make observations twice daily for a month, in others for four or five days consecutively. In all I have made about 2000 observations, carried out at all hours of the day and night. In each I was careful to use only Kew-tested and corrected thermometer, allowing rather more than the full time of exposure by a watch, as I have frequently observed that ordinary thermometers vary very much (some being about $\frac{4}{10}$ths or $\frac{6}{10}$ths too high or too low at certain temperatures).

"Besides my own observations I am indebted to work which has been done by the late Professor Robertson, Mr. Hugh Singleton, Mr. George Armatage, Mr. Fleming, Mr. Stapley, and Mr. William Robertson, also to records of foreign observers, among whom are such well-known names as Colin, Siedamgrotsky, Krabbe, and Wesley Mills.

"I find that many conditions alter considerably the normal temperatures. That in all animals the temperature is slightly lower when they have been at rest for some time than when taken immediately after work. In the evening it is usually from a fourth to an eighth of a degree higher than in the morning, being lowest about 4 to 5 A.M., when the animals have been undisturbed for a considerable time. In the fowl and duck, however, the contrary (as might be expected) is the rule; their temperature being highest from about 10 to 12 midday, and lowest at night when they have been at rest and quiet for some time.

"Feeding causes a rise of temperature, and well-fed animals are always higher than poorly kept ones. In hot weather I have thought that the temperature was slightly higher than in cold weather. I suppose that tissue-metabolism is greater. The temperature of the vagina is generally about $\frac{2}{10}$ths under that of the rectum; but in taking the two, one after the other, if the animal be excitable, it is possible almost always with a two-minute thermometer to get the last one taken equal to or greater than the first.

"Exercise and excitement each tend to send the temperature up very quickly. There is, however, a limit to the rise of the mercury, beyond which no further excitement will cause it to go. I have made numerous experiments solely to watch this. The time taken, too, for the temperature to come down again is considerable, often almost two hours.

"In confinement the temperature is generally a little less than when the animal is at liberty. This is especially noticeable in dogs, sheep, and pigs. Young animals are always higher than old ones.

"The horse in health, when at rest, varies from 99° to 101°; at work I have frequently observed it higher. I have placed the average at 100.2°.

"For the cow, dog, and cat I have placed the average at 101.4°. I have frequently sent the dog's temperature up 2° in twelve minutes by sharp exercise, and, even by merely causing him to struggle for three or four minutes (as when placed on an operating table) I have observed it to rise 0.4 and 0.6 of a degree.

"The pig varies very much, this animal is so difficult to catch without excitement and struggling. I have, however, managed to get a number very quietly, especially when feeding. The average here mentioned, 102.6°, considers an adult pig to be from nine to eighteen months old. Old sow pigs will be as low as 100° before food and exercise, and young pigs, if raced about for only a few minutes before securing, as high as 106°. Feeding, too, makes a very great difference to this animal; more so, I think, than to any other.

"In the case of the sheep the same remarks apply, though not quite to such an extent. In confinement a sheep varies from 102.9° or so to about 104°, but when at liberty from 103.2° to 104.8°. Exercise and excitement soon sent them up to 105° and higher.

"The fowl and duck are very easily excited and can readily be induced to show a temperature of 109°, and in one case I twice observed 111° in a healthy duck. Fowls after being at rest for some time range from 105.5° to 107°, but after being at liberty from 106° to 108°, the duck rather higher than the fowl.

"I have also made observations on the ostrich, goat, and other creatures, but did not consider them of sufficient utility to insert here."—Reported in the *Veterinary Record*, December 28, 1895.

THE SIXTH INTERNATIONAL VETERINARY CONGRESS IN BERNE, September 16 to 21, 1895. In our January number we were only able to give the report of the Congress up to the close of September 18th. We are happy now to add a summary of the discussions and resolutions for the remaining days.

The session of September 19th was given up to a discussion of the best means of fighting the swine-plague. A written report entitled "The War on the Swine-plague" was presented by Director Zschokke, of Zurich. Professor Preiss (of Buda-Pest) delivered his report, and after a long discussion the following resolution of Director Zschokke was adopted almost unanimously. "Both epidemics, which are designated as swine-plague and swine-pest are, for practical reasons, to be entered under one common name in the epidemics which are to be fought by the State; they are to be placed under the law requiring that cases be officially reported and are to be entered in the epidemic bulletins apart from bacterial erysipelas. Further methods of fighting the disease will be best regulated by the separate countries in accordance with their laws and the local conditions."

In regard to the worth of inoculation as a preventive against erysipelas in swine opinions differ widely. After a long debate the following motion of Dr. Lorenz (from Darmstadt) was adopted almost unanimously: "Preventive inoculation is an indispensable means in fighting erysipelas among swine."

On the other hand, a second resolution of Dr. Lorenz was accepted by only a small majority: "This Congress takes occasion to call the attention of the Government to, and to recommend that they, by the granting of means, support the application of inoculation as a preventive against erysipelas in swine, and that they, by a supervision of the inoculations and establishment of statistics, demonstrate the value of the different methods of inoculation."

The next subject on the programme was the discussion of inoculation against madness in dogs, both as a preventive and a cure. Dr. Pourtalé, together with Professor Jolyet (of Bordeaux), made a written report in which he stated, as a result of his investigations, that the poison causing madness entirely loses its virulence after it has been weakened by being transferred to goats several times. In the discussion it soon came to light that inoculation in the case of hydrophobia had a rather small number of supporters. After a short debate the following pretty, colorless resolution was adopted: "Congress expresses the wish that all who are interesetd in experimental investigations will verify the facts which lie at the basis of the results offered in the report in order to pave the way for the introduction of preventive inoculation against rabies in the different European States."

A long report was then made by Dr. Lydtin, of Carlsruhe, on the fourth subject on the programme, namely: The influence of veterinary science on the social development and on the elevation of the well-being of a people. The question was further explained by the author and gave no occasion to discussion or to a resolution.

The discussion of No. 5 on the programme, namely, lung disease among cattle and the report of the results of the regulations adopted in different countries in order to stamp it out, was not brought to a conclusion at the session of September 19th, and was postponed until the following day. In regard to the war upon lung disease (tuberculosis), there were several very thorough and interesting reports. For the United States, Dr. Liautard (New York); for Holland, Denmark, Luxemburg, and Belgium, Thomassen (Utrecht); for the German Empire, Roeckel (Berlin); for France and England, Leblanc (Paris); for Italy, Generali (Modena); for Austria, Sperk (Vienna); for Sweden and Norway, Lindquist (Stockholm); for Switzerland, Hirzel (Zurich); and for Spain and Portugal, Vrurrun y Rodriguez (Madrid). A report in regard to the spread of the disease in Russia was not given, and Professor Persu, of Bukarest, declared that so far the outbreak of tuberculosis had not been observed in Roumania. The very thorough discussion which followed showed that the preponderating majority of the members declared themselves in favor of fighting the disease by killing all animals which are either sick with the disease or suspected of it. This view found expression in the following resolution, in favor of which several resolutions had been withdrawn during the course of the debate, only two voting against it: "The Congress is of the opinion that the infectious lung-plague among cattle can be in a short time completely hindered in its spread if the following regulations are observed in fighting it: *a.* Animals which are sick with tuberculosis should be forever excluded from public traffic of every kind. *b.* As soon as the disease breaks out it is necessary to kill all infected animals as well as those suspected of being infected. *c.* Compulsory meat-inspection should be introduced everywhere." In fighting the lung-plague only a small value was laid upon preventive inoculation. Advantage from the same can only be expected under certain conditions, such as exist, for instance, in the so-called Spoeling District, in the Netherlands, and at present in the Prussian District of Magdeburg. The Congress adopted, as an addition to the resolution named above, the following motion of Arloing: "Preventive inoculation against tuberculosis can only be of service in infected herds where the stock is often renewed and the effectiveness of sanitary regulations is more or less limited by persistent and unavoidable conditions." During the discussion it was explained that inoculation against tuberculosis had been undertaken only under special conditions which had been established by the authorities, and that no one was allowed to dispense inoculin (vaccine) without the consent of the authorities, as it cannot be intrusted to everybody.

In the session of September 20th, after the discussion in regard to tuberculosis had been concluded, the question of the utilization of the flesh of tuberculous animals and its relation to public health came up. Three printed reports formed the basis of discussion: Butel (of Meaux), "Tuberculous Meats and Public Hygiene;" De Jong (of Leyden), on the "Sterilization of Tuberculous Meat;" Guillebeau (of Berne), "The Utilization of the Flesh of Tuberculous Animals and the Public Health." The discussion on this subject was a very animated one because many, especially the French members, were of the opinion that all animals which were found to be afflicted with tuberculosis should be excluded from all use as animals fit for slaughter without regard to how wide the disease had spread in the body of the animal under consideration. The members from Roumania declared that this practice had been carried out in their land with great strictness.

Butel, who in his report had in general held the same view, favored in the course of his discussion the following resolution, which was offered by several members and carried by a large majority: 1, the flesh (meat) of tuberculous animals is to be subjected to especial regulations; 2, if in the carrying out of these regulations the meat must be confiscated, the owners are to be proportionately indemnified in so far as they have observed the sanitary provisions; 3, meat is to be seized as soon as tuberculous changes through their spread or character cause the same to appear dangerous; 4, meat is to be excluded from all traffic if it comes from an emaciated animal; if it has a bad appearance; if tubercular changes are found in the muscular tissues; if tubercular changes of an essential nature are present on several of the intestines; 5, it is to be desired that tuberculous meat which has been declared fit for use will be put upon sale in certain designated markets with a statement as to its origin after it has been subjected to an effective mode of sterilization; 6, the Congress utters the wish that the erection of sterilizing apparatus may be aided as much as possible on the part of the governments. General conclusions: The Congress calls the attention of the governments of the States officially represented to the necessity of introducing obligatory meat-inspection. Of the numerous other motions only that of Director Trasbot (Alfort, Paris) was almost unanimously adopted. The other motions were partly voted down, partly not put to vote, and partly withdrawn in favor of Dr. Trasbot's resolution, which was as follows: "The Congress expresses the wish that in every land a commission may be established with the object of clearly defining those cases in which the inspectors of public and private slaughter-houses are to make a total confiscation of the meat when the presence of tuberculosis is established by a section (cutting)."

At the conclusion of the session the report of the commission was heard which had deliberated upon the question of establishing a uniform anatomical nomenclature. The conclusions reached can be summed up briefly in the statement that the "Nomenclator Anatomicus," which was published at the beginning of 1895, and which has been adopted as authority by physicians, is to be the authority for veterinary anatomy also. The names as far as it is necessary, under the supervision of a commission consisting of a German, French, and Belgian anatomist, are to be adapted to the needs of veterinary anatomy, and the result is to be adopted once for all at a meeting of the members of the commission one year before the next International Veterinary Congress meets.

The final session of September 21st was devoted to determining the time and place of the next International Veterinary Congress, and decided that the same should be held in 1899 in Baden-Baden, provided the Grand Ducal government approved the decision. The organization of the Congress is left to the members of the present Congress from Baden, with power to coöperate with other members if necessary.

The proceedings of the Congress were taken by a stenographer and the publication of the report will follow during the winter.

The members of the Congress eagerly seized the opportunity to visit the Veterinary School in Berne, which has been entirely remodelled, and had a chance to become acquainted with the latest arrangements which have been made to meet all the demands of science and practice. The professors in Berne showed every kindness to the visitors. Opportunity was further given on this occasion to obtain more exact knowledge of the changes which tuberculosis brings about in cattle. This had in one case been shown to such an extremely small degree that only the result of a tuberculin inoculation, as Professor Nocard demonstrated, confirmed the suspicion that the animal was infected with tuberculosis. Professor Degive operated upon two cryptorchids, in which in the first case one testicle and in the second both had not descended into the scrotum. He explained these operations in a long address to the members. The Agricultural Exposition taking place at the same time in Berne gave opportunity to become acquainted with the finest breeds of cattle in Switzerland.

The final session of the Congress took place in Interlaken, whither the members had been carried by a special train and steamer, which was placed at their disposal by the Swiss Federative Council. With this meeting a dinner provided by the same Coucil was connected, and a great number of toasts were drank in which all gave expression to their feelings of gratitude for the charming hospitality of Switzerland. The hearty intercourse between members of the most different nationalities will always remain an object of pleasant recollection.—Müller: *Archiv für Wissenschaftliche und praktische Thier. Heilkunde*, Band xxii. Heft 1 u. 2.

Examination of the Rectum for Diagnostic Purposes. By Svend Larsen, Assistant in the Veterinary High School in Copenhagen. (*Monatsheft für praktische Thierheilkunde.*)

The inflation of the smaller intestine with air is generally caused as well by the shifting of its position, as, for example, in the twisting of its axis or in the pressing in of the Winslow aperture, as by a stoppage (obstruction) in the smaller intestine. In all these cases an inflated knot in the smaller intestine can be felt by rectal examination, about the shape of sausage and as thick as one's arm. Several of these inflated twists in the intestine are sometimes found, and they lie generally a little in front of the entrance to the pelvis, usually toward the right. The cause of the inflation cannot, as a rule, be determined by an examination of the rectum. A stoppage in the small intestine can easily be felt, and is readily distinguished from a knot or ball of feces in the rectum. In special cases, however, it should be possible to make a diagnosis of obstruction in the small intestine, as a stoppage, consisting of a sausage-like, doughy body of 8 cm. in diameter, can be felt. If a single inflated knot in the smaller intestine, which stands out prominently and lies lengthwise along the wall of the stomach, can be felt, it points to a pressing in of Winslow's aperture. A lipoma (fatty tumor), with a handle-like excrescence, cannot be easily felt between the distended knots of the smaller intestine. In most cases the distention by the air is probably caused by the shifting of position in the intestine, and this, in connection with the other symptoms, forms an important aid in making the diagnosis.

Sometimes in cases of colic a change in the position of the spleen can be felt without any apparent cause. Under normal conditions the spleen is felt on the left side pretty far toward the front and close to the wall of the stomach. Here the sharp back edge can be felt, and, as a rule, a small part of the surface which faces the middle of the stomach. If the spleen, as mentioned, can be pushed aside, it takes place so that it can be pushed partly further back into the cavity of the stomach, or, what is more striking, partly because its back edge can be separated a little from the wall of the stomach, and feels like a sharp edge standing vertically, which, on being pressed, can be pushed to one side, but which immediately assumes its original position when the finger is withdrawn. It is capable of being pushed aside so far that the fist can easily be inserted between the wall of the stomach and the back edge of the spleen. What causes this shifting of position in the spleen is a question which has not yet been answered; it probably arises in connection with some obstruction (certainly, as in cases of colic), but the obstruction cannot be felt, which is hardly to be expected, since a stoppage which would shove the spleen out of place must lie very far toward the front. It is most natural to think here of overflowing of the stomach, besides it is not impossible that a stoppage in the stomach, like enlargement of the larger intestine, could cause such a shifting. A case which came under my observation not along ago points to the fact that shifting of the spleen can take place, however, without being caused by a stoppage. A horse which was suffering from glanders was, during its last days, examined repeatedly per rectum, because it was feared that it had metastases (transferrence of disease from one part to another) in the abdomen (bowels, lower part of the belly). The last examination was made a few hours before the horse was killed. At every examination the spleen was shoved toward the back and so far toward the middle that there was ample room for the fist between it and the wall of the stomach. The post-mortem showed neither stoppage nor anything abnormal in the hinder part of the body.—*Thierärzt Centralblatt*, December 1, 1895.

Sterilizing Hair Used in Manufacture to Prevent Splenic Fever and Anthracoid Diseases. At the session of the Lower Austrian Sanitary Council held on November 18, 1895, a report of the Institute of Hygiene in the University of Vienna was read in regard to the results of the bacteriological investigation made upon specimens of hair, which were regarded as the source of infection for several cases of splenic fever (anthracoid diseases), which had occurred in the businesses of the brush manufacturers and the spinners of horse-hair. The institute had made several experiments for determining the best method of sterilization.

The Sanitary Council declared first of all the existence of anthracoidal spores which were found in the places where hair was handled could be taken as an unquestioned proof that they were the cause of the disease in the cases mentioned above. In regard to the precautionary measures recommended by the Hygienic Institute, the Sanitary Council declared, further, that the same demanded the introduction of a series of sanitary regulations affecting the spinneries of horse-hair, the brush manufacturers, and similar businesses, and also obligatory disinfection of hair before it was worked up. It thought, however, that these improvements in regard to the handling of the hair would only have a palliative worth and that a complete eradication of anthracoidal infections in the manufactures mentioned could only be obtained by obligatory disinfection of the raw wares, whether this be effected by means of the action of ordinary steam or by the steam (or vapor) of formaldehyde, which had been found to produce an immediate effect by the Hygienic Institute.

The council felt compelled to recommend the establishment of a central disinfecting institute in order to carry out either the one or the other of these means.—*Thierärzt Centralblatt*, December 15, 1895.

